# Aggressive Follicular Lymphoma of the Breast: An Unusual Suspect

**DOI:** 10.7759/cureus.91770

**Published:** 2025-09-07

**Authors:** Kabeer Ali, Christopher Mikulas, Austin Quan, Tasnuva Rashid, Walter JR Quan

**Affiliations:** 1 Internal Medicine, University of Florida College of Medicine – Jacksonville, Jacksonville, USA; 2 Pathology and Laboratory Medicine, University of Florida College of Medicine – Jacksonville, Jacksonville, USA; 3 Hematology/Oncology, University of Florida College of Medicine – Jacksonville, Jacksonville, USA

**Keywords:** breast lymphoma, follicular lymphoma, follicular lymphoma of the breast, non-hodgkin lymphoma, pbl, primary breast lymphoma

## Abstract

Follicular lymphoma (FL) of the breast represents an uncommon subtype of breast lymphoma, an unusual type of non-Hodgkin lymphoma (NHL). It typically presents as a secondary disease, meaning it is a manifestation of systemic lymphoma involving the breast rather than a primary breast origin. While screening efforts and outcomes of patients with breast cancer have risen dramatically in the last few decades, the same cannot be said of breast lymphoma. This can be partly due to the rarity of the condition and also due to the difficulty in diagnosing it. The diagnosis can often be masked by other conditions, such as benign breast masses or more common breast malignancies, such as carcinoma. This can lead to delays in diagnosis and disease progression. We describe a case of a patient who was diagnosed with aggressive FL of the breast. Despite the usual indolent nature of the disease, the patient’s complicated medical comorbidities, including coronary artery disease (CAD), chronic obstructive pulmonary disease (COPD), and diabetes, resulted in challenges in treatment.

## Introduction

Non-Hodgkin lymphoma (NHL) is the 11^th^ most common cancer worldwide and the 11^th^ leading cause of cancer-related death, with an estimated 570,000 to 590,000 new cases and between 270,000 and 280,000 deaths reported in 2024, reflecting a rising trend [1, 2]. In the United States, over 80,000 new cases of NHL are projected in 2024, according to the Surveillance, Epidemiology, and End Results (SEER) database [3]. NHL encompasses a diverse group of malignancies arising from lymphoid tissue, including both indolent and aggressive subtypes. Established risk factors for NHL include immunodeficiency disorders, HIV infection, obesity, hypertension, diabetes, advancing age, female sex, and family history [1]. NHL can be classified into two main types: nodal and extranodal lymphoma. Nodal lymphoma originates in lymphatic tissues, including the lymph nodes, spleen, pharyngeal lymphatic ring, and thymus. Extranodal lymphoma originates in any organ outside the lymphatic system. Common extranodal sites include the gastrointestinal system, central nervous system, and skin. The gastrointestinal tract is the most common extranodal site, accounting for up to 20% of all extranodal NHL [4, 5]. 

Follicular lymphoma (FL), a subtype of NHL, is the second most common form of lymphoma in the United States. It accounts for approximately 35% of NHL cases and 70% of low-grade lymphomas [6]. The median age at diagnosis is 65 years [6]. Primary breast lymphoma (PBL) is a rare manifestation of extranodal NHL, representing roughly 1% of all NHL cases and less than 0.5% of breast malignancies. The majority of PBL cases are diffuse large B-cell lymphoma (DLBCL), with a minority comprising follicular or marginal zone lymphoma subtypes. This is generally associated with a poorer prognosis compared to indolent subtypes, such as follicular or mucosa-associated lymphoid tissue (MALT) lymphoma [5]. Given its rarity, management of primary breast FL is not well-defined, so it is often treated the same as FL in other locations. For limited-stage disease (stage I and II), involved-field radiotherapy (IFRT) is the first-line treatment with consideration of rituximab [7]. For advanced-stage disease (stages III and IV), monitoring is recommended in asymptomatic patients with low tumor burden. In symptomatic patients with high tumor burden, first-line therapy includes induction therapy with rituximab in combination with chemotherapy [8]. Recommended chemotherapy regimens include bendamustine or cyclophosphamide, vincristine, doxorubicin, and prednisone (CHOP) versus cyclophosphamide, vincristine, and prednisone (CVP) for patients not fit for CHOP.

## Case presentation

**Figure 1:**
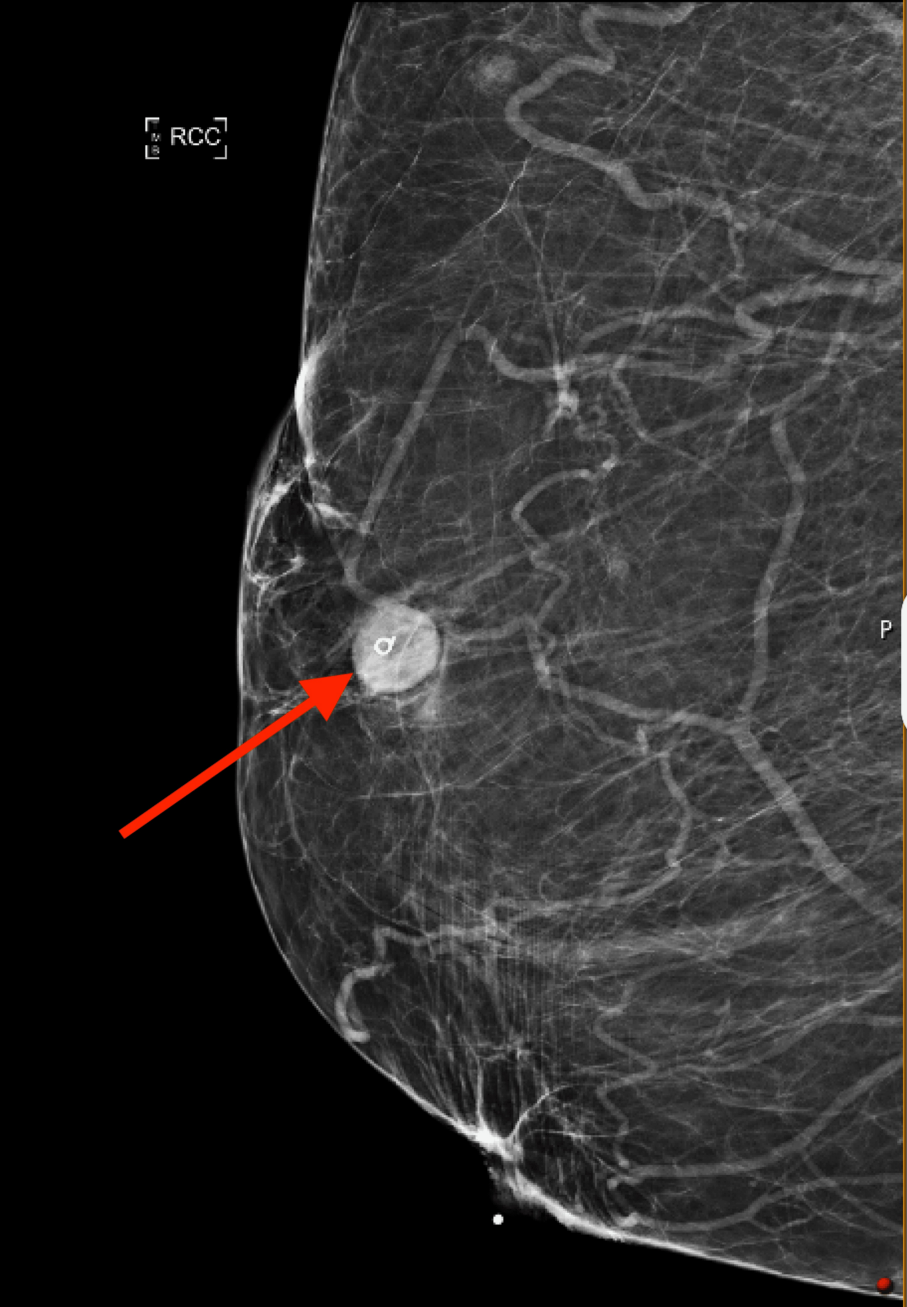
Mammogram with arrow demonstrating an oval-shaped, circumscribed, hypoechoic mass measuring 1.9 x 1.7 centimeters (cm) within the upper outer right breast, approximately 9.5 cm from the nipple (Breast Imaging-Reporting and Data System 2 (BI-RADS-2)).

A 59-year-old female with a past medical history significant for type 1 diabetes mellitus, chronic obstructive pulmonary disease (COPD), and coronary artery disease (CAD) presented to the hematology-oncology clinic for a new breast mass. She had also been diagnosed with hypertension, hyperlipidemia, stage 2 chronic kidney disease, class 3 obesity, and obstructive sleep apnea. The patient first noticed her symptoms two months before the visit. She described a “pulling” sensation in her right breast when reaching for an object that brought her attention to a lump. She denied any B-symptoms, including significant weight loss, fever greater than 100.4 degrees Fahrenheit, or drenching night sweats. A diagnostic mammogram showed an oval-shaped, circumscribed, hypoechoic mass measuring 1.9 x 1.7 centimeters (cm) within the upper outer right breast, approximately 9.5 cm from the nipple, categorized as Breast Imaging-Reporting and Data System 2 (BI-RADS-2) (Figure 1). No suspicious calcifications, architectural distortion, or skin thickening were seen.

**Figure 2:**
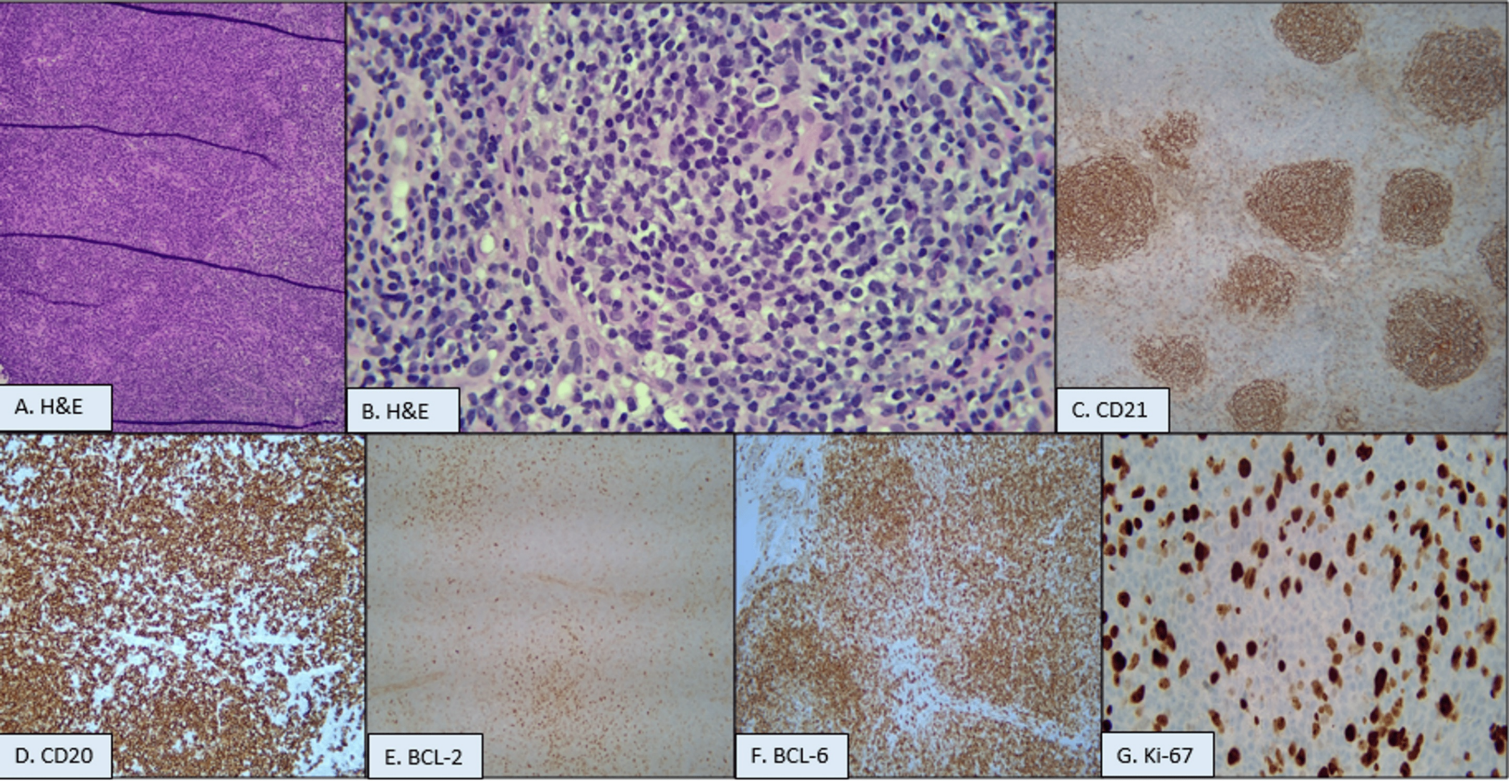
Pathological images from breast core needle biopsy with various immunohistochemistry markers and Ki-67. A. 4X; B. 20X; C. 10X; D. 20X; E. 20X; F. 20X; and G. 20X magnifications

An ultrasound-guided core needle biopsy of her right breast was performed and revealed an atypical lymphoid infiltrate. Immunohistochemistry (IHC) was significant for positive cluster of differentiation-20 (CD20), BCL2, and BCL6; negative BCL1, CD3, CD5, CD30, and cyclin D1; and variable Ki67 proliferation index up to 50% to 60% (Table 1, Figure 2). Fluorescence in situ hybridization (FISH) was requested for dual specificity phosphatase-22 (DUSP22)-interferon regulatory factor-4 (IRF4) rearrangement, which was not detected. These findings were consistent with grade 2 FL. The Ki67 proliferation index was concerning for a higher-grade FL. Thus, complete staging with positron emission tomography (PET) scan and bone marrow aspiration and biopsy was ordered.

**Table 1:** Breast lesion core needle biopsy results and serum laboratory values with reference ranges for comparison

Laboratory value	Patient value	Reference range
Immunohistochemistry markers		
CD20		N/A
BCL2		N/A
BCL6		N/A
BCL1		N/A
CD3		N/A
CD5		N/A
CD30		N/A
Cyclin D1		N/A
Ki-67 expression	60%	0-3%
Hemoglobin	13.5 g/dL	12.0-15.5 g/dL
Lactate dehydrogenase	242 IU/L	119-214 IU/L

**Figure 3:**
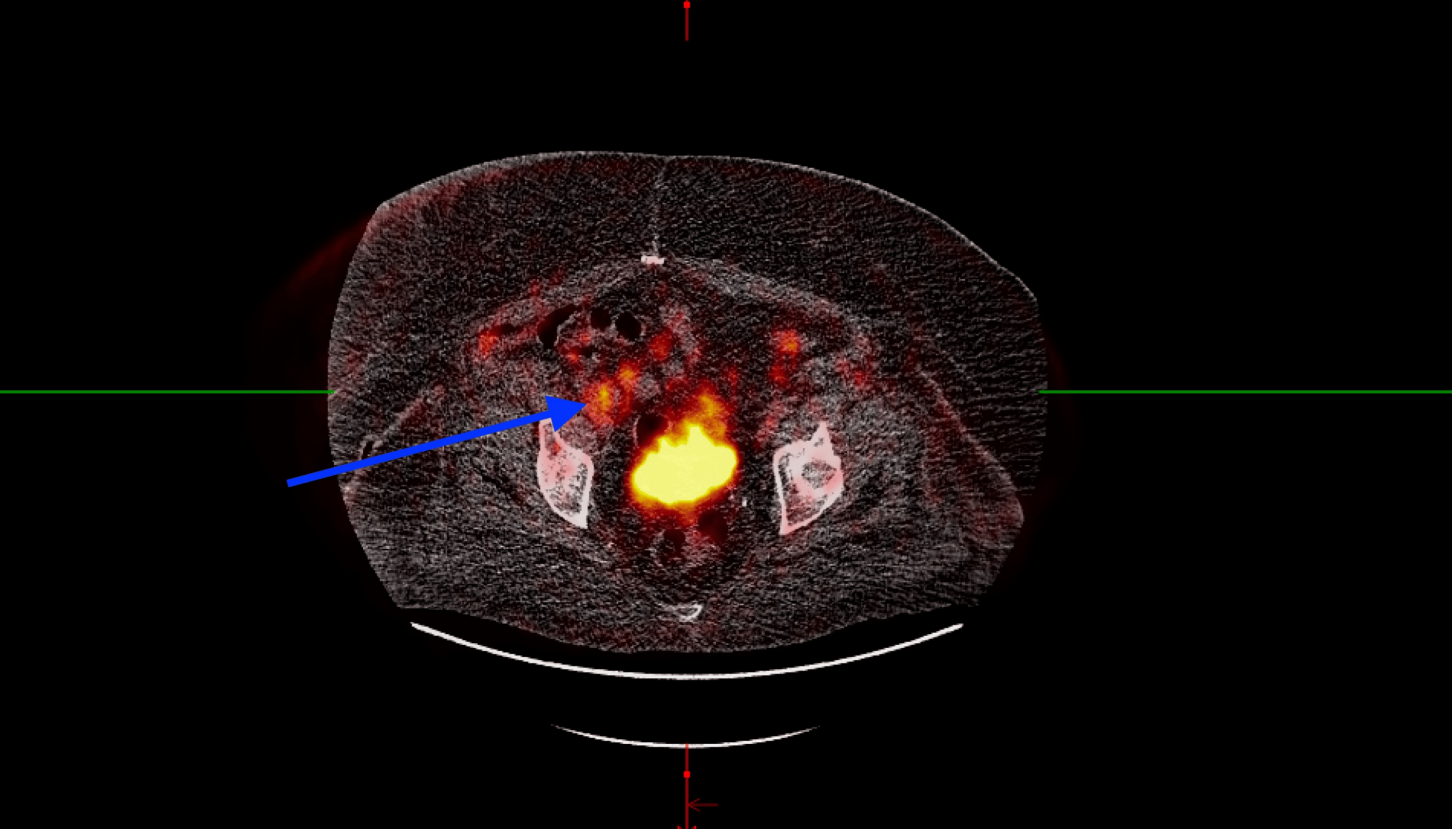
Positron emission tomography (PET) scan with an arrow showing a new fluorodeoxyglucose (FDG)-avid lymph node within the right inguinal region measuring 1.9 x 3.7 x 2.4 cm with a maximum standardized uptake value (SUV) of 5.10, indicating increased metabolic activity associated with cancerous growths, but can also be seen in inflammation or infection.

The PET scan revealed a fluorodeoxyglucose (FDG)-avid lymph node within the right inguinal region measuring 1.9 x 3.7 x 2.4 cm (Figure 3). Bone marrow biopsy and aspirate showed no morphologic evidence of involvement by acute leukemia, lymphoma, or high-grade myelodysplasia. Flow cytometry showed no overt immunophenotypic abnormalities, and cytogenetic analysis demonstrated a normal female phenotype in all cells. The workup was otherwise significant for a hemoglobin of 13.5 grams (g)/deciliter (dL) and lactate dehydrogenase (LDH) of 242 international units (IU)/L. The final diagnosis after complete staging was primary breast FL, grade II, stage IIIa. The International Prognostic Score (IPI) was two, correlating with a low-intermediate risk group.

Treatment with bendamustine was initiated with a plan to treat for a maximum of three cycles to palliate symptoms with a goal of cytoreduction in view of tumor burden, given histologically and immunophenotypically confirmed FL. After three cycles and subsequent CT scans, rituximab every eight weeks for up to 12 doses would be intended. The patient, unfortunately, required two admissions in three months before initiating chemotherapy. The first admission was for non-ST-segment elevation myocardial infarction when the patient was found to have severe multivessel CAD and deemed a poor surgical candidate for coronary artery bypass grafting. The second admission was for a COPD exacerbation. Given severe multivessel CAD, frequent congestive heart failure (CHF) and COPD exacerbations, and impaired mobility along with hypertension and hyperlipidemia, it was decided to proceed with chlorambucil rather than bendamustine, given its lower toxicity profile and better tolerability for systemic treatment for six cycles with CT chest, abdomen, and pelvis after cycle 2.

During cycle three, the patient presented to the emergency department for purulent drainage from a lesion of her right breast. She was diagnosed with cellulitis, and admission for intravenous (IV) antibiotics was recommended. However, the patient refused admission, so she was discharged with a seven-day course of oral amoxicillin/clavulanic acid and doxycycline. On follow-up with hematology-oncology, the patient reported persistent but improved purulent drainage from the right breast cellulitis with three days remaining of the antibiotic course. Thus, chlorambucil was held due to acute infection, and a right breast ultrasound was ordered, which revealed no abscess.

One month later, the patient was admitted to the intensive care unit for acute hypoxic respiratory failure requiring intubation and mechanical ventilation due to a CHF exacerbation. A CT of the chest showed no disease progression of her lymphoma. Her admission was complicated by septic shock due to ventilator-acquired pneumonia. The patient was extubated but ultimately became dependent on noninvasive positive pressure ventilation. Given the patient’s history of multiple intubations and concerns regarding excessive dynamic airway collapse based on previous bronchoscopy findings, she was considered a candidate for tracheostomy, to which she consented. The patient ultimately suffered a cardiac arrest secondary to hypoxia from mucus plugging in her tracheostomy despite appropriate suctioning and tracheostomy care, with a return of spontaneous circulation after advanced cardiac life support. Unfortunately, this was complicated by anoxic brain injury. The lymphoma burden remained stable throughout the hospitalization. After comprehensive goals-of-care discussions with the family, a decision was made to transition to comfort care only. The patient subsequently passed away shortly thereafter.

## Discussion

FL is the second most common lymphoma in the United States. It accounts for about 35% of NHL and 70% of low-grade, or indolent, lymphomas. FL presents in extranodal locations in approximately 10% of cases [6]. However, primary breast FL is an infrequent entity. Only 0.3% to 0.7% of NHLs are PBLs, most of which are DLBCLs [9]. The aggressiveness of the malignancy increases with an increase in the number of centroblasts; however, less than 20% of patients present with B symptoms [10]. Indolent FL exhibits slow clinical progression and limited genetic complexity. In contrast, transformed FL presents with aggressive clinical features and additional genetic alterations associated with high-grade behavior and a poor prognosis [11]. Approximately 85% of FLs will demonstrate a t(14;18), which results in the overexpression of the BCL-2 protein that blocks apoptosis [12]. FL most commonly presents as painless lymphadenopathy affecting the axillary, femoral, cervical, and inguinal lymph nodes [13]. Bone marrow involvement occurs in approximately 70% of cases [13]. PBL most often presents as a painless, enlarging mass. Skin retraction, erythema, and peau d’orange suggest high-grade disease. Nipple retraction and discharge, common in breast carcinomas, are uncommon in PBL [14]. Our patient’s presentation, marked by a painless breast mass without B symptoms or nipple changes, was consistent with these typical features. The absence of bone marrow involvement placed her in the minority of FL cases.

In this case, the patient presented with a breast lump, making our patient’s described location particularly notable [15]. FL is most commonly an indolent process with a favorable prognosis, with estimated 10-year survival rates ranging from 72.4% to 86.6% [16]. Two-year overall survival (OS) rates are 98%, 94%, and 87% for low-, intermediate-, and high-risk groups, respectively [13]. Notably, patients with primary breast FL may have inferior progression-free survival (PFS) and OS compared to those with limited-stage nodal FL, suggesting a poorer prognosis associated with breast involvement [10]. Given the aggressive disease course of this patient, the question should raise suspicion for future patients observed with diagnosed FL with unusual primary sites. Another point of discussion from the diagnostic process of this disease is the potential to misdiagnose breast lymphoma as breast carcinoma. PBL can mimic breast carcinoma, especially in limited tissue samples from core needle biopsies, and lead to delays in appropriate treatment. The use of IHC and molecular analysis is crucial for accurate diagnosis. Breast carcinoma remains a major focus in the media and scientific research, and breast lymphoma may be misdiagnosed as breast carcinoma due to anchoring bias and premature closure. The incidence of PBL increased significantly from 1975 to 1999 [17]. Patients with follicular PBL tend to have inferior PFS and OS compared to those with limited-stage nodal follicular NHLs, especially in those with poor baseline functional status, such as this patient [8]. Radiographically, PBL on mammography often appears as a large, well-circumscribed or ill-defined mass without microcalcifications or spiculations. Lesions tend to be larger than typical breast carcinomas (4-5 cm vs. 2-3 cm) and are less likely to show architectural distortion. While ultrasound and MRI can support evaluation, their imaging features are nonspecific and cannot reliably distinguish PBL from other malignancies [18]. In our case, mammography revealed a hypoechoic, circumscribed lesion without calcifications or distortion. Interestingly, the lesion was smaller (1.9 × 1.7 cm) than typical PBL tumors, and no axillary lymphadenopathy was present. IHC plays a critical role in diagnosing FL and distinguishing it from other breast neoplasms. FL characteristically expresses CD19, CD20, CD10, and BCL6 and is negative for CD5 and CD23. Nearly all cases overexpress BCL2 due to the hallmark t(14;18) translocation [11]. Our patient’s biopsy confirmed CD20, BCL2, and BCL6 positivity and CD5 negativity, consistent with FL. Histologically, FL recapitulates the germinal centers of secondary lymphoid follicles. Neoplastic cells are a mix of small- to medium-sized centrocytes and larger centroblasts in the germinal center of secondary follicles. The number of centroblasts per high-power field (hpf) determines the histologic grade: grade I (<5 centroblasts/hpf), grade II (6-15/hpf), and grade III (>15/hpf). Grade III is further subdivided into IIIa (centrocytes present) and IIIb (sheets of centroblasts) [13]. Our patient’s pathology showed 6-15 centroblasts/hpf, consistent with grade II FL.

The Ann Arbor staging system is commonly used to classify NHL. Stage I involves a single lymph node region; stage II includes two or more regions on the same side of the diaphragm; stage III involves nodes on both sides of the diaphragm; and stage IV denotes disseminated extranodal involvement. Each stage is further categorized by the presence (B) or absence (A) of systemic symptoms [19]. PET-CT is the preferred imaging modality for staging and response assessment in FL. Our patient was classified as stage IIIa, with involvement of both the right breast and right inguinal lymph node on the PET scan, but no B symptoms or extranodal organ involvement. The Follicular Lymphoma International Prognostic Index (FLIPI) includes five risk factors: age >60, stage III/IV disease, involvement of >4 nodal sites, elevated serum LDH, and hemoglobin <12 g/dL. Patients are stratified as low (0 to one risk factors), intermediate (two risk factors), or high risk (≥3 risk factors) [13]. With stage III disease and an LDH of 242 IU/L, our patient had two risk factors, placing her in the intermediate-risk category. High tumor burden, as defined by the French Groupe d’Etude des Lymphomes Folliculaires (GELF) criteria, includes any of the following: tumor mass >7 cm, ≥3 nodal sites >3 cm, B symptoms, splenomegaly, compression syndrome, serous effusions, leukemic phase, or cytopenias [20]. Our patient met none of these criteria and was considered to have a low tumor burden.

Due to its rarity, there is no standard treatment algorithm for primary breast FL, and management typically mirrors that of FL at other sites. For limited-stage (I-II) disease, radiotherapy with or without rituximab is often preferred. For advanced-stage (III-IV) disease, asymptomatic patients with low tumor burden may be monitored, while symptomatic or high-burden patients are treated with rituximab-based chemoimmunotherapy. Regimens include bendamustine with rituximab (BR) or CHOP ± rituximab. Less intensive combinations, such as CVP, are options for frail patients [8]. While the standard approach to advanced-stage FL often involves concurrent administration of bendamustine and rituximab, our patient was treated with a sequential approach, beginning with three cycles of bendamustine monotherapy for induction, followed by rituximab monotherapy as maintenance. This approach reflects a palliative strategy aimed at balancing disease control and treatment tolerability. Pegfilgrastim was used to mitigate bendamustine-associated myelosuppression [21]. Although limited data exist on this exact sequence, the use of delayed rituximab has precedent in maintenance strategies, such as that shown in the Primary Rituximab and Maintenance (PRIMA) trial, which demonstrated PFS benefit with rituximab maintenance following induction in FL [22]. Due to progressive cardiac and pulmonary comorbidities and poor functional status, the patient ultimately transitioned to chlorambucil, a less intensive alkylating agent generally reserved for elderly or frail patients. While chlorambucil monotherapy produces lower response rates than rituximab-based regimens, it is a reasonable option for those unable to tolerate aggressive therapy due to its favorable toxicity profile [23].

Our patient’s FL showed no radiographic progression during her final hospitalization, signifying that her mortality was not lymphoma-related. Her ultimate decline and death were instead the result of complications from her extensive comorbid conditions and not directly as a consequence of lymphoma treatment. These comorbidities significantly limited the patient’s treatment options and contributed to recurrent hospitalizations, infectious complications, and eventual respiratory failure. This case underscores the importance of individualized treatment planning that considers both the malignancy and the broader clinical context in which it occurs. While FL generally carries a favorable prognosis, concurrent chronic illness can substantially alter outcomes and limit therapeutic possibilities. The broader implications for elderly patients with indolent lymphoma who have multiple comorbidities include a significantly increased risk of non-lymphoma-related mortality, reduced overall survival, and a higher likelihood of treatment-related toxicity.

## Conclusions

This case highlights a rare presentation of FL of the breast and underscores the challenges in managing uncommon extranodal manifestations. Accurate diagnosis is crucial, as the treatment for lymphoma differs significantly from that of breast carcinomas, involving chemotherapy and radiotherapy rather than surgical intervention. Despite an early diagnosis, the patient’s disease course was significantly influenced by comorbid conditions, underscoring the complexity of care in such patients. PBL should be included in the differential diagnosis for any atypical breast lesion, particularly in patients with systemic symptoms or risk factors for lymphoproliferative disorders. Treatment modification due to comorbidities and diagnostic complexity may be necessary.
